# Longitudinal autoantibody responses against tumor-associated antigens decrease in breast cancer patients according to treatment modality

**DOI:** 10.1186/s12885-018-4022-5

**Published:** 2018-01-31

**Authors:** Rick L. Evans, James V. Pottala, Satoshi Nagata, Kristi A. Egland

**Affiliations:** 1 Cancer Biology Research Center, Sanford Research, Sioux Falls, SD USA; 2 Sanford School of Medicine, University of South Dakota, 2301 East 60th Street North, Sioux Falls, SD 57104 USA; 3 Center for Drug Design Research, National Institutes of Biomedical Innovation, Health and Nutrition, Ibaraki-City, Osaka 5670085 Japan

**Keywords:** Breast cancer, Autoantibodies, Tumor-associated antigens, Treatment modalities

## Abstract

**Background:**

Metastatic breast cancer (BCa) is most often diagnosed months after completion of treatment of the primary tumor when a patient reports physical symptoms. Besides a physical examination, no other alternative recurrence screening method is recommended for routine follow-up care. Detection of autoantibodies against tumor-associated antigens (TAAs) has demonstrated promise for distinguishing healthy women from patients diagnosed with primary BCa. However, it is unknown what changes occur to patient autoantibody levels during and after treatment.

**Methods:**

Three serial blood draws were collected from 200 BCa patients: before treatment, 6 and 12 months after surgery. Patients were categorized according to treatment regimen, including surgery, chemotherapy, radiation, trastuzumab and hormonal therapies. The longitudinal samples were assayed for autoantibody responses against 32 conformation-carrying TAAs using a Luminex multiplex bead assay.

**Results:**

The treatment modality groups that had the greatest decrease in autoantibody response levels were radiation + hormonal therapy; radiation + chemotherapy; and radiation + hormonal therapy + chemotherapy. For these three treatment groups, autoantibody responses against 9 TAAs (A1AT, ANGPTL4, CAPC, CST2, DKK1, GFRA1, GRN, LGALS3 and LRP10) were significantly reduced at 12 months after surgery compared to before treatment. One TAA, GRP78, had a significantly increased autoantibody response after 12 months.

**Conclusions:**

Single treatment regimens alone did not significantly alter autoantibodies levels against the studied TAAs. Radiation treatment was the common denominator of the three most affected groups for significant changes in autoantibody response levels.

**Electronic supplementary material:**

The online version of this article (10.1186/s12885-018-4022-5) contains supplementary material, which is available to authorized users.

## Background

Early diagnosis of breast cancer (BCa) is critical for increased disease survival, both at the time of initial disease as well as for recurrence [1, 2]. Including all races, the 5-year survival rate for women diagnosed with local BCa is 99%. Survival declines to 84% for regional stage and plummets to 26% for distant stage [3–5]. Approximately 6–10% of U.S. women present with metastatic BCa when first diagnosed [5–7], and 20 to 50% of patients initially diagnosed with primary BCa will develop metastatic disease [7]. Incurable metastatic disease is responsible for most BCa-related deaths. Unfortunately, recurrence of BCa is most often found when patients report symptoms, such as shortness of breath, chronic cough, headache, weight loss or bone pain. Once a patient is diagnosed with metastatic BCa, the intent for treatment is no longer curative; instead, the goal is to control the disease for as long as possible [8, 9]. Presently, no alternative screening methods are recommended for asymptomatic patients without clinical findings on physical examination [10]. By the time the metastases are identified by physical symptoms, the patient’s chances of surviving the disease are greatly diminished [11]. Lack of early detection of recurrence for asymptomatic patients is a factor in the inability to cure metastatic BCa.

Numerous previous studies have detected the presence of autoantibodies to various cancer proteins in BCa patients [12, 13]. Measurement of these autoantibodies could offer early detection of BCa recurrence before physical symptoms are apparent, which would have a monumental impact by giving patients the choice of appropriate treatment options at an earlier stage of disease [14]. We previously screened patient plasma samples for autoantibodies against 20 different tumor-associated antigens (TAAs) and compared the profiles to healthy women utilizing an ELISA-based platform. The 20 antigens were selected from a membrane-associated polyribosomal cDNA library (MAPcL), which encodes membrane and secreted proteins highly expressed in BCa and should preferentially induce an antibody response in patients [15]. The conformation of membrane and secreted proteins is particularly important because discontinuous epitopes will only be present for antibody recognition when the antigen is folded properly. Expression constructs were generated to encode the extracellular portion of the TAA fused to rabbit Fc (rFc), and a eukaryotic expression system was developed to produce conformation-carrying antigens that are processed with post-translational modifications [16]. A panel of 7 conformation-carrying TAAs, consisting of ANGPTL4, DKK1, LGALS1, MUC1, GFRA1, GRN and LRRC15, was identified that in combination could discern newly diagnosed BCa patients from healthy controls with a 73% sensitivity and 76% specificity [16]. To continue in the development of this technology, the single-well ELISA-based autoantibody assay was transitioned to a multiplex Luminex platform. The Luminex xMAP microsphere technology (Luminex, Austin, TX) allows measurement of the interaction of patient autoantibodies with a panel of antigen biomarkers enabling quantitation across all biomarkers in a single well [17].

It is unknown what, if any, changes occur to a BCa patient’s autoantibody profile after the tumor is removed and treatment begins. Because the autoantibodies specifically recognize cancer antigens, the autoantibody response may decrease during and after treatment due to the reduced tumor burden in the body. To address this question, the multiplex bead assay was performed on serial blood draws of 200 newly diagnosed BCa patient samples. Autoantibody responses against 32 antigens were determined over the course of both time and treatment, including surgery, radiation, chemotherapy, antibody and hormonal therapies.

## Methods

### Plasmid construction and protein production

Details for construction of plasmids encoding rabbit Fc (rFc)-tagged tumor-associated antigens (TAAs) have been described previously [16]. Briefly, the extracellular domain of transmembrane proteins or the full-length sequence of secreted and intracellular proteins was cloned into pSecTag2-rabbit Fc or pFUSE-rFc1. These plasmids included a secretion signal, as well as a C-terminal rFc tag. The TAA-rFc plasmids were transfected into 293 T cells (catalogue # CRL-3216, ATCC, Manassas, VA) with Effectene (Qiagen, Valencia, CA), and the encoded proteins were secreted into the cell culture supernatant. Supernatants were harvested after transfection, and TAA-rFc content was measured with an anti-rFc sandwich ELISA.

### Antibody coupling to Luminex xMAP magnetic beads

Goat anti-rabbit IgG Fc antibody (Jackson Immunoresearch, West Grove, PA) was coupled to Luminex xMAP beads utilizing the xMAP AbC Kit (Luminex, Austin, TX) according to the manufacturer’s instructions. Briefly, beads were activated with EDC (1-Ethyl-3-[3-dimethylaminopropyl]carbodiimide hydrochloride) and Sulfo-NHS (*N*-hydroxysulfosuccinimide) for 20 min. After washing with phosphate buffered saline (PBS, pH 7.4), anti-rFc antibody was added to the beads at a concentration of 20 μg per 1 × 10^6^ beads and incubated for 2 h shielded from light. The beads were washed again and stored at 4 °C shielded from light until use. Coupling was performed on 32 Luminex bead regions that can be mixed for use and distinguished in 32 different areas by a Luminex 100/200 instrument (Millipore).

### Loading of tumor-associated antigen-rabbit Fc fusion proteins to Luminex beads

Beads coupled with the anti-rFc antibody were coated with each TAA-rFc fusion protein by incubation with the cell culture supernatant containing secreted TAA-rFc fusion protein. Each of the 32 TAA-rFc fusions was bound to one Luminex bead region. Beads were incubated with the fusion protein at 40 μg/10^6^ beads overnight at 4 °C. Beads were stored in PBS-TBN buffer (PBS with 0.1% bovine serum albumin, 0.02% Tween 20 and 0.05% sodium azide) at 4 °C in the dark until use.

### Patients

Patients for this study were recruited from Sanford Health, Sioux Falls, SD. Patients newly diagnosed with any type of BCa and were 30 years of age or older were invited to participate. A 10 ml EDTA tube of blood was collected from each of 200 patients prior to mastectomy, lumpectomy, chemotherapy, radiation therapy, or other treatment (Table 1). The patient descriptions and collection methods have previously been described elsewhere [16]. Patients enrolled in the study were followed for 1 year after surgery, and blood draws were obtained at follow-up oncology visits at 6 and 12 months after surgery. Potential subjects were excluded from the study if they had been previously diagnosed with any type of cancer. The longitudinal blood samples were collected from 10/08/09 to 8/27/13. The Sanford Health IRB approved the clinical protocol and consent forms for this study. Written informed consent was obtained from all patients before participation.

**Table 1:** Patient clinical and pathological characteristics

Patients with Breast Cancer	*N* = 200
Age: Mean (SD)	58.9 (11.4)
White Race: n (%)	193 (97%)
BMI [kg/m2]: Mean (SD)	29.7 (6.6)
*Smoking Status: n (%)*	
Current	22 (11%)
Never	120 (60%)
Past	58 (29%)
Family History Yes: n (%)	114 (58%)
*Tumor Type: n (%)*	
Invasive	148 (74%)
in situ	52 (26%)
*Tumor Max Dimension [cm]: n (%)*	
≤ 1	66 (36%)
> 1 to ≤2	65 (35%)
> 2	53 (29%)
*Histology: n (%)*	
Ductal and Lobular	3 (2%)
Ductal	173 (87%)
Lobular	21 (11%)
Other	2 (1%)
ER Positive: n (%)	171 (86%)
PR Positive: n (%)	147 (74%)
*HER2 Amplification: n (%)*	
Negative	132 (66%)
Positive	15 (8%)
Unknown	53 (27%)
Triple Negative: n (%)	18 (12%)
Lymph Node Involvement: n (%)	47 (24%)
Matched Healthy Controls	N = 200
Age: Mean (SD)	58.8 (11.3)
White Race: n (%)	192 (97%)
BMI [kg/m2]: Mean (SD)	27.1 (5.5)
*Smoking Status: n (%)*	
Current	7 (4%)
Never	125 (63%)
Past	67 (34%)

### Plasma collection and storage

The 10 ml EDTA tube was centrifuged at 2000 × *g* for 10 min within 12 h of drawing. Plasma was collected as the supernatant, placed in aliquots and stored at − 80 °C until the assay for the autoantibodies.

### Multiplex autoantibody bead assay

Autoantibodies against the 32 TAAs in the plasma samples were measured simultaneously in a single well utilizing a multiplex bead assay. The xMAP Luminex magnetic beads from 32 distinct regions, each coated with a different TAA-rFc fusion, were combined and distributed to the wells of a 96-well round bottom plate. Plasma samples from BCa patients were diluted 1:10 in FACS buffer (PBS with 5% fetal bovine serum and 0.1% sodium azide), and 200 μl of diluted plasma was applied to the beads in a single well. Samples were incubated with beads for 2 h on ice. Beads were then pelleted magnetically and washed twice, with a final aspiration leaving only the beads. As the secondary antibody, R-Phycoerythrin (PE)-labeled goat anti-human IgG (Jackson Immunoresearch, West Grove, PA) was diluted 1:200 in FACS buffer and 200 μl was added to the beads of each well. After an incubation of 1 h on ice, beads were washed twice. The beads in each well were re-suspended in 200 μl of FACS buffer and analyzed on a Luminex 100/200 instrument, with a minimum of 100 events analyzed for each bead region. Each plate included a secondary only negative control, as well as a PE goat anti-rabbit IgG (Jackson Immunoresearch, West Grove, PA) reacting with bead-loaded TAA-rFc fusions as a positive control. All washing and aspiration steps were performed with a Biotek ELx405 microplate washer with magnetic capabilities.

### Statistical methods

Each 96-well plate had a negative control *background* and a positive control *standard* for all autoantibodies. In addition, each patient had her baseline, 6 and 12 month samples analyzed in the same 96-well plate. For each autoantibody, the patient’s median fluorescent intensity (MFI) value had its plate MFI background level subtracted and was then shifted by the minimum constant to make all values at least one. The value was then normalized by the ratio of the MFI for the standard over the mean standard MFI across all 8 plates. Lastly the values were log transformed to stabilize the variance and produce a more symmetric distribution, i.e. autoantibody response = LN[(autoantibody – background + constant)*(standard – background)/mean(standard – background)]. To measure precision, the inter-assay CV for each TAA was calculated across the plates for the positive and negative controls.

A repeated measure ANOVA was used to model the geometric mean changes from baseline for each autoantibody over time (i.e. 6 and 12 months) as an exploratory analysis, with a compound symmetry correlation structure. The models included all interactions among indicator variables for radiation, hormonal and chemotherapy with time. This approach included 183 out of 200 BCa patients in the primary analysis; the 17 patients given trastuzumab as part of their treatment consisted of 4 groups and were analyzed secondarily by examining their mean response profile. Point estimates and 95% confidence intervals (CI) were calculated for all 8 treatment combinations, which did not include trastuzumab, at 6 and 12 months in order to rank autoantibodies by the number of positive findings. Since each TAA had 16 (8 treatments at 6 and 12 months) point estimates tested, one false positive was expected for each TAA; therefore, the observed geometric mean changes were presented for autoantibody levels with 3 or more positive findings. A *p*-value < 0.05 was used to ascribe statistical significance, and SAS (Cary, NC) version 9.3 was used for all analyses.

## Results

Serial plasma samples were collected from 200 newly diagnosed BCa patients [16]. Characteristics of the 200 BCa patients enrolled in this study have been previously described by our laboratory, including demographic information, tumor size, tumor marker status, in situ versus invasive components and lymph node involvement (Table 1) [16]. Blood draws were acquired before treatment, 6 and 12 months after surgical resection of the primary tumor. To determine the patients’ autoantibody responses against cancer antigens over the course of treatment, 32 TAA-rFc fusion proteins consisting of 20 previously analyzed TAAs [16] and 12 newly selected cancer antigens were generated (Table 2). The initial 20 antigens were selected from the MAPcL, which encodes membrane and secreted proteins highly expressed in BCa with minimal expression in normal tissues [15]. The additional 12 antigens were selected based on previous literature demonstrating an autoantibody response against the cancer protein, regardless of the protein localization [18–29]. For consistency, the eukaryotic expression system developed previously [16] was used to generate all of the TAA-rFc antigens for the multiplex immunoassay. Thirty-two sets of Luminex beads consisting of unique red/infrared emission spectra were coated with anti-rabbit IgG. The 32 TAA-rFc fusion proteins were attached to the coated Luminex beads followed by incubation with plasma samples acquired from patients before treatment, 6 and 12 months post surgery. The average inter-assay CV for the 32 autoantibody responses measured at baseline (before the start of treatment) with the Luminex multiplex bead platform were 11.1 and 11.4% for the low and high controls, respectively (Additional file 1).

**Table 2:** Tumor-associated antigens for generation of rFc fusion proteins

Gene	Accession #	Signal Sequence Amino Acids	Encoded Amino Acid Fragment
20 Original Antigens			
ANGPTL4	NM_139314	1–30	31–406
CD147	NM_198589	1–21	22–162
CD320	NM_016579	1–46	47–230
CDH3	NM_001793	1–24	25–654
CST2	NM_001322	1–20	21–141
DKK1	NM_012242	1–28	29–266
EPHA2	NM_004431	1–26	27–535
GFRA1	AF038421	1–24	25–465
GRN	NM_002087	1–17	18–593
ERBB2	NM_004448	1–22	23–652
IGFBP2	NM_000597	1–39	40–328
LAMC2	NM_005562	1–21	22–1111
LGALS1	NM_002305	1–17	18–135
LRP10	NM_014045	1–16	17–440
LRRC15	NM_001135057	1–27	28–544
MUC1	NM_002456	1–22	23–167
SPINT2	NM_021102	1–27	28–198
SPON2	NM_012445	1–26	27–331
SSR2	NM_003145	1–17	18–146
SUSD2	NM_019601	1–27	28–785
12 Additional Antigens			
A1AT	NM_000295.4	1–24	25–418
AMACR	NM_014324.5	None	1–382
BIRC5	NM_001168.2	None	1–142
CALD1	NM_033139.3	None	1–558
CAPC	NM_001013653.2	1–26	27–264
CCNB1	NM_031966.3	None	1–433
CCND1	NM_053056.2	None	1–295
GRP78	NM_005347.4	None	1–654
LGALS3	NM_002306.3	None	1–250
MYC	NM_002467.4	None	1–439
NY-ESO-1	NM_001327.2	None	1–180
XAGE1	NM_001097594.2	None	1–81

Each subject was categorized based on the treatments received during the 12 months following initial diagnosis. All enrolled BCa patients in our study underwent surgery to remove the primary tumor, including a breast lumpectomy or mastectomy. In addition to surgery, treatment included combinations of hormonal therapies, trastuzumab, radiation and cytotoxic chemotherapy (Table 3). Seventy-two patients received cytotoxic chemotherapy consisting of one of the following regimens: adriamycin/cytoxan + taxol = 33 patients; cytoxan + taxol = 22 patients; adriamycin/cytoxan = 5 patients; carboplatin + taxol = 6 patients; adriamycin + cytoxan/taxol = 3 patients; adriamycin/cytoxan + carboplatin/Gemzar = 1 patient; carboplatin + taxotere + novantrone = 1 patient; taxol alone = 1 patient. If a patient had any one of the chemotherapy regimens described above, they were considered part of the chemotherapy treatment group. Grouping the study participants based on treatment regimen resulted in 12 groups, ranging from no treatment after surgery to all treatment modalities administered (Table 3). Eight of the 12 groups received a combination of at least two therapies in addition to surgery. Three groups received a single therapy in addition to surgery, and one group received surgery alone.

**Table 3:** Number of patient blood draws per treatment modality and visit

Treatment Group^a^	Baseline	6 Month	12 Month	Subtotal
Radiation + Hormonal	59 (29.5%)	52	52	163
Hormonal	31 (15.5%)	25	26	82
Radiation + Hormonal + Chemotherapy	25 (12.5%)	23	22	70
Surgery Only	24 (12.0%)	15	10	49
Hormonal + Chemotherapy	15 (7.5%)	12	13	40
Radiation	13 (6.5%)	8	8	29
Radiation + Chemotherapy	11 (5.5%)	11	11	33
Radiation + Hormonal + Chemotherapy + Trastuzumab	8 (4.0%)	8	8	24
Chemotherapy	5 (2.5%)	4	5	14
Hormonal + Chemotherapy + Trastuzumab	5 (2.5%)	5	5	15
Radiation + Chemotherapy + Trastuzumab	3 (1.5%)	2	2	7
Radiation + Hormonal + Trastuzumab	1 (0.5%)	1	1	3
Total number of samples	200 (100%)	166	163	529

^a^All patients received surgery to remove the primary tumor

**Fig. 1:**
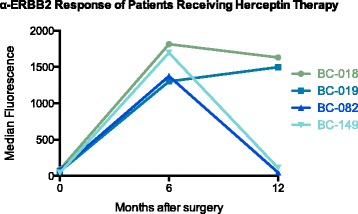
Representative antibody responses against ERBB2-rFc in patients diagnosed with ERBB2 positive breast cancer treated with Trastuzumab. Three longitudinal blood draws were collected, immediately before surgery, 6 and 12 months after surgery. Patients BC-018 and BC-019 were still receiving Trastuzumab therapy at their 12-month visit, and patients BC-082 and BC-149 discontinued Trastuzumab therapy prior to their 12-month visit

Four of the 12 treatment groups received trastuzumab therapy (17 patients total, Table 3). Trastuzumab is a humanized monoclonal antibody that is generally administered every 3 weeks for 1 year to patients with amplification of the *ERBB2* gene [30]. Since ERBB2 is one of the TAAs included in our assay, the treatment of these patients with trastuzumab provided a fortuitous internal spiked control. We determined the signal levels of the serial blood draws against ERBB2 for the 17 patients. Two main response patterns were observed correlating with time of trastuzumab administration. First, for patients that had discontinued trastuzumab therapy after 6 months, their anti-ERBB2 antibody responses for the 12-month blood draws decreased (Fig. 1, represented by patients BC-082 and BC-149). The second pattern is represented by patients BC-018 and BC-019 in Fig. 1. These patients continued to receive trastuzumab during the longitudinal blood draws, and the anti-ERBB2 antibody response against the ERBB2 antigen plateaued between the 6 and 12-month visits (Fig. 1).

For the primary exploratory analysis, we excluded the 17 patients who received trastuzumab as part of their therapy. Therefore, the analysis included 183 out of 200 patients and encompassed 8 of 12 patient treatment groups (Table 3). A repeated measure ANOVA was used to model the geometric mean changes from baseline for each autoantibody at 6 and 12 months, and the model included all interactions among indicator variables for radiation, hormonal therapy and chemotherapy with time. If the ANOVA model predicted at least 3 significant changes for an antigen among the 16 estimates (8 groups * 2 time points consisting of 6 and 12 month blood draws), it was chosen for further analysis. Using this variable selection criterion, the model identified 11 antigens that were likely to have significantly modulated autoantibody signals in response to treatment in the 12 months following surgery. The TAAs chosen for further study included: A1AT, ANGPTL4, CAPC, CST2, DKK1, GFRA1, GRN, GRP78, LGALS3, LRP10 and NY-ESO-1.

**Fig. 2:**
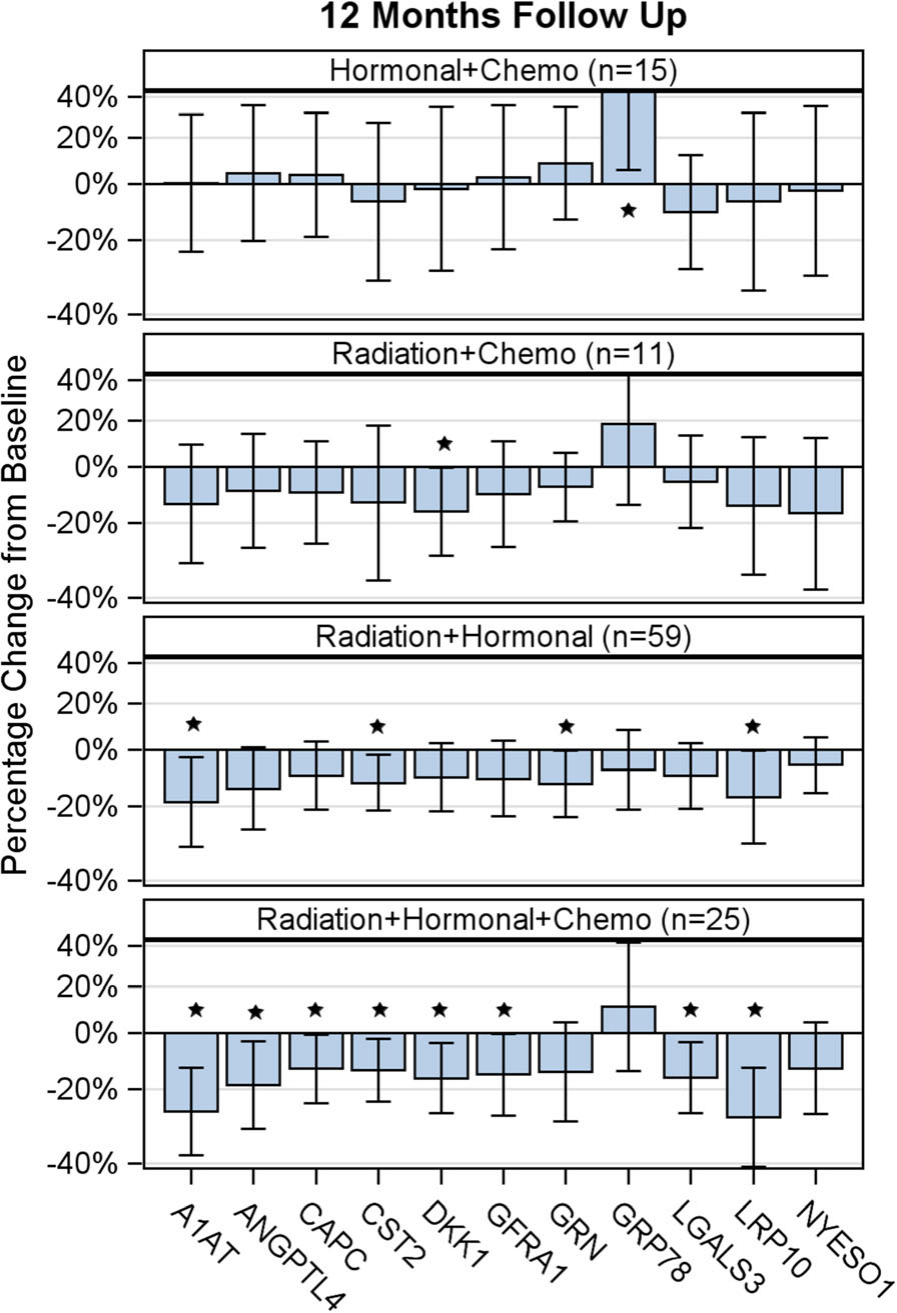
Observed geometric mean changes of autoantibody responses at 12 months after baseline. The graph indicates the observed geometric mean changes (with 95% confidence intervals) of autoantibody levels against 11 tumor-associated antigens according to treatment regimen. Changes were calculated between baseline (before start of treatment) and 12 months after the start of treatment. * indicates *p*-value < 0.05. There were no significant changes observed for surgery only or individual therapies, including hormonal, radiation or chemotherapy

The actual geometric mean changes from baseline were calculated for the 11 TAAs at 6 months (Additional file 2) and 12 months (Fig. 2). No significant changes in the autoantibody response against the 11 antigens were observed for patients who received surgery alone or were treated with surgery and a single monotherapy: hormonal therapy, chemotherapy or radiation. While NY-ESO-1 was predicted to have significant changes in levels of response in the repeated measures ANOVA model, the observed changes were not significant (Fig. 2). However, significant decreases in levels of response between baseline and 12 month time points against 9 of the 11 TAAs (i.e. A1AT, ANGPTL4, CAPC, CST2, DKK1, GFRA1, GRN, LGALS3 and LRP10) were observed in three treatment groups. Radiation + chemotherapy, radiation + hormonal therapy, and radiation + hormonal therapy + chemotherapy had average geometric mean decreases for the 9 significant TAA of − 11, − 13, and − 18%, respectively (Fig. 2). In the radiation + hormonal therapy + chemotherapy group, A1AT and LRP10 had the greatest decrease in autoantibody response levels (− 26 and − 28%, respectively) compared to the other antigens and regimens. The triple therapy of radiation + hormonal + chemotherapy was more effective at reducing the autoantibody responses against the TAAs than any other combination of treatment. In addition, the decrease in response of the geometric mean was − 15% at 6 months after surgery (Additional file 2) compared to a greater decrease of − 18% at the 12-month time point (Fig. 2). It is apparent that autoantibody levels diminish during the course of treatment, and they continue to decrease with time as the patient is further removed from initial diagnosis. At the 12-month time point, GRP78 was the only TAA to exhibit a significantly increased autoantibody response of 44%, which occurred in the hormonal therapy + chemotherapy group (Fig. 2).

## Discussion

Measurement of autoantibody responses to cancer proteins holds promise as a minimally invasive screening method in cancer diagnostics and prognostics. Numerous TAAs that elicit an immune response have been identified, and findings in this realm continue to expand [31–33]. Although studies have shown the presence of the autoantibodies at the time of BCa diagnosis [13, 34, 35], little is known about how these antibodies are maintained over time and affected by treatment. This study provides important data on longitudinal changes of autoantibody response occurring in BCa patients after surgery. We were able to assess 32 TAAs simultaneously with our unique multiplex bead assay. In addition, rich data on pathology and treatment information allows the findings of this study to be associated with the treatment regimens administered to each patient.

Patients in this study that were *ERBB2*-amplified received trastuzumab treatment, which recognizes and binds to a native pocket-like binding region of ERBB2 [36, 37]. As predicted, the levels of trastuzumab against ERBB2 in patient plasma correlated temporally with patient treatment (Fig. 1). To remove the confounding variable of trastuzumab treatment, patients treated with the anti-ERBB2 antibody were removed from the primary analysis. The data collected from the longitudinal blood draws indicated that significant changes in antibody responses were detected at the 6-month time point (Additional file 2), with a more extensive reduction detectable 12 months after the primary tumor was surgically resected and therapy initiated (Fig. 2). The absence of a large tumor mass is one explanation for the reduced production of autoantibodies to TAAs. With the majority of the cancer cells removed, the immune system would be exposed to fewer cancer antigens reducing the levels of autoantibody production. Alternatively, the decrease in autoantibody activity could be due to radiation + chemotherapy and/or hormonal therapy-induced immunosuppression. The fact that a single treatment modality after surgery, including radiation alone, did not alter the levels of autoantibodies indicates that it is not simply due to treatment-induced immunosuppression.

Surprisingly, we also found that treatments administered after the removal of the primary tumor had a profound effect on the autoantibody profile. GRP78 showed an increase in autoantibody signal at the 12-month time point in patients receiving chemotherapy followed by hormonal therapy. This protein is typically localized to the endoplasmic reticulum [38], but it has been shown to translocate to the cell surface in cancer cells [39], particularly in times of cellular stress [40]. The cellular stress imposed on tumor cells by anti-neoplastic treatments would explain a change in localization of GRP78 from the endoplasmic reticulum to the surface, making it more available to the immune system in its aberrant location. Production of anti-GRP78 antibodies could occur in this situation, explaining the outlying antibody increase obtained for this TAA.

There is one confounding factor that is difficult to overcome when attributing the data to the treatment groups: the treatment given to a patient is dictated by the physical characteristics of the tumor, i.e. the presence of the estrogen receptor, amplification of *ERBB2*, size of the tumor, in situ versus invasive components and lymph node involvement. The changes seen in these samples are attributed to the therapies administered to that patient, but it is acknowledged that these therapies are a function of the characteristics of each subtype of BCa [41, 42].

To that end, the treatment modality groups that had the greatest decrease in autoantibody response levels were radiation + hormonal therapy; radiation + chemotherapy; and radiation + hormonal therapy + chemotherapy (Fig. 2). The common denominator of the three most affected groups for significant changes in autoantibody response levels is radiation treatment. However, radiation treatment alone is not enough to significantly decrease the response levels of the autoantibodies (Fig. 2).

Four antigens (ANGPTL4, DKK1, GFRA1 and GRN) overlapped with the ability to discern BCa patients from healthy [16] and to elicit an autoantibody response against them that was significantly lowered over the course of treatment (Fig. 2). Yet, three of the seven previously characterized antigens (LGAL1, LRRC15 and MUC1) were not altered longitudinally in the current study. It is possible that these antigens only have utility in the initial diagnosis of BCa and do not exhibit a statistically significant reduction in autoantibody levels after treatment. Another explanation is that the 7 antigens utilized to discern health from cancer patients were analyzed as a group and not individual responses against each antigen.

The ultimate goal of detecting autoantibodies in the follow-up setting is to determine if the autoantibody response to a panel of cancer antigens can predict patient response to treatment and detect recurrence of the disease. Future studies will include acquiring serial blood draws before and after the patient presents with physical symptoms of recurrence. The fact that the autoantibody levels of BCa patients decrease over the course of treatment when radiation is used in combination treatment modalities supports the potential of using the detection of these autoantibody levels as a prognostic indication of recurrence.

## Conclusions

Adjuvant treatment following the removal of the primary tumor had an extensive effect on the autoantibody profile of the patient. Autoantibody levels of BCa patients against TAAs decreased over the course of treatment when radiation was used in combination treatment modalities. Using a Luminex-based multiplex bead analysis, significant decreases in levels of response against A1AT, ANGPTL4, CAPC, CST2, DKK1, GFRA1, GRN, LGALS3 and LRP10 were observed between baseline and 12 month time points in three treatment groups: Radiation + chemotherapy, radiation + hormonal therapy, and radiation + hormonal therapy + chemotherapy. A significant increase in autoantibody response was observed against GRP78 in the hormonal therapy + chemotherapy group.

## Additional files

Additional file 1: Table indicating inter-assay coefficients of variability (CV) for the Luminex multiplex immunoassay. Shown are the average inter-assay CV for the autoantibody responses against the 32 TAAs for negative and positive controls. Calculations were measured at baseline (before the start of treatment) using the Luminex multiplex bead platform. (DOCX 32 kb)

Additional file 2: Observed geometric mean changes of patients’ autoantibody responses at 6 months after the start of treatment. The graph indicates the observed geometric mean changes (with 95% confidence intervals) of autoantibody levels against 11 tumor-associated antigens according to treatment regimen after 6 months follow-up. *indicates *p*-value < 0.05. There were no significant changes observed for surgery only or individual therapies (i.e. hormonal, radiation, or chemotherapy). (DOCX 117 kb)

## Abbreviations

BCa: Breast cancer
CI: Confidence interval
ELISA: Enzyme-linked immunosorbent assay
MAPcL: Membrane-associated polyribosomal cDNA library
MFI: Median fluorescence intensity
PBS: Phosphate buffered saline
PE: R-Phycoerythrin
rFc: Rabbit Fc
TAA: Tumor-associated antigen
