# Supplementary material for: Longitudinal autoantibody responses against tumor-associated antigens decrease in breast cancer patients according to treatment modality
Source: BMC Cancer. 2018 Jan 31;18:119. doi: 10.1186/s12885-018-4022-5 (PMC5793406; doi:10.1186/s12885-018-4022-5)
Supplement: Supplementary file 1 — Table indicating inter-assay coefficients of variability (CV) for the Luminex multiplex immunoassay. Shown are the average inter-assay CV for the autoantibody responses against the 32 TAAs for negative and positive controls. Calculations were measured at baseline (before the start of treatment) using the Luminex multiplex bead platform. (DOCX 32 kb) [file 12885_2018_4022_MOESM1_ESM.docx]

**Additional File 1. Table of Inter-assay coefficient of variation for negative**

**and positive controls for 32 TAA on the Luminex bead platform.**

| **Autoantibody** | **Negative Control**  **(Background)** | **Positive Control (Standard)** |
| --- | --- | --- |
| **Original 20** | | |
| ANGPTL4 | 9.2 | 12.3 |
| CD147 | 5.7 | 10.7 |
| CD320 | 21.8 | 12.9 |
| CDH3 | 7.5 | 12.3 |
| CST2 | 13.4 | 12.2 |
| DKK1 | 6.6 | 9.7 |
| ERBB2 | 9.3 | 12.2 |
| EPHA2 | 5.2 | 12.6 |
| GFRA1 | 9.6 | 11.5 |
| GRN | 11.7 | 15.4 |
| IGFBP2 | 14.7 | 9.7 |
| LAMC2 | 7.7 | 11.1 |
| LGALS1 | 14.7 | 11.7 |
| LRP10 | 5.5 | 10.3 |
| LRRC15 | 5.7 | 16.1 |
| MUC1 | 8.3 | 10.1 |
| SPINT2 | 12.9 | 9.2 |
| SPON2 | 14.3 | 10.5 |
| SSR2 | 18.2 | 11.2 |
| SUSD2 | 7.0 | 12.3 |
| **Additional 12** | | |
| A1AT | 12.2 | 12.8 |
| AMACR | 11.3 | 11.2 |
| BIRC5 | 12.8 | 11.3 |
| CALD1 | 11.8 | 9.9 |
| CAPC | 10.2 | 10.4 |
| CCNB1 | 12.8 | 9.2 |
| CCND1 | 10.9 | 10.3 |
| GRP78 | 11.9 | 11.7 |
| LGALS3 | 13.1 | 13.9 |
| MYC | 12.5 | 9.5 |
| NY-ESO-1 | 15.9 | 11.2 |
| XAGE1 | 11.3 | 10.9 |
| **Average** | 11.1 | 11.4 |
