# Supplementary figures and images for: Longitudinal autoantibody responses against tumor-associated antigens decrease in breast cancer patients according to treatment modality
Source: BMC Cancer. 2018 Jan 31;18:119. doi: 10.1186/s12885-018-4022-5 (PMC5793406; doi:10.1186/s12885-018-4022-5)

**
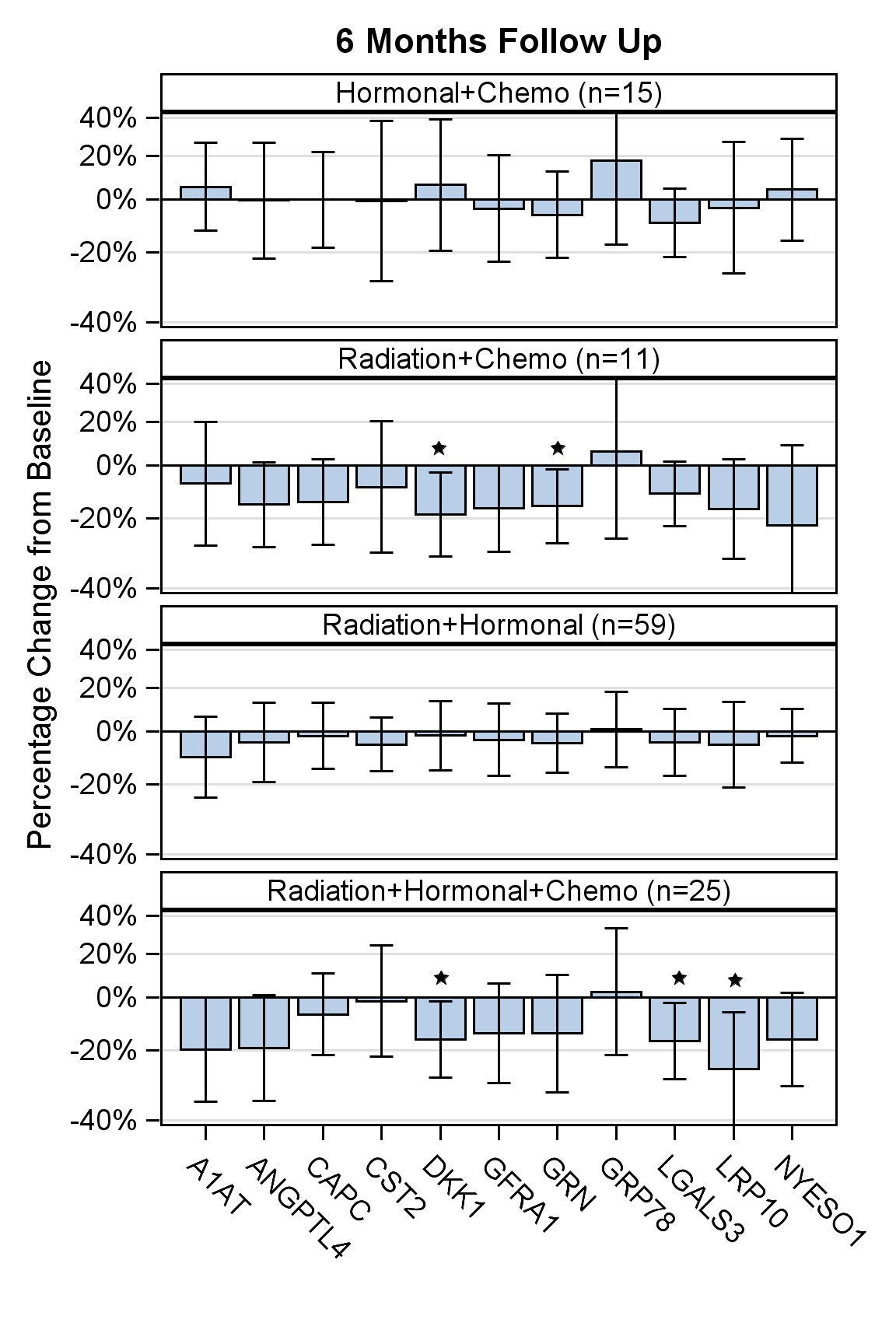
**

**Additional File 2**

Supplement: Supplementary file 2 — Observed geometric mean changes of patients’ autoantibody responses at 6 months after the start of treatment. The graph indicates the observed geometric mean changes (with 95% confidence intervals) of autoantibody levels against 11 tumor-associated antigens according to treatment regimen after 6 months follow-up. *indicates p-value < 0.05. There were no significant changes observed for surgery only or individual therapies (i.e. hormonal, radiation, or chemotherapy). (DOCX 117 kb) [file 12885_2018_4022_MOESM2_ESM.docx]
